# Lytic properties and genomic analysis of bacteriophage Brt_Psa3, targeting *Pseudomonas syringae* pv. *actinidiae*

**DOI:** 10.1007/s00253-025-13613-z

**Published:** 2025-10-11

**Authors:** Emil Gimranov, Hugo Oliveira, Conceição Santos, Luísa Moura, Joana Azeredo

**Affiliations:** 1 Centre of Biological Engineering, University of Minho, Campus de Gualtar Braga, Braga, 4710-057 Portugal; 2 Biology Department, Faculty of Science, University of Porto (FCUP), Porto, 4169-007 Portugal; 3 LAQV-REQUIMTE, Biology Department, Faculty of Science (FCUP), University of Porto, Porto, 4169-007 Portugal; 4 CISAS—Center for Research and Development in Agrifood Systems and Sustainability, Instituto Politécnico de Viana Do Castelo, Rua Escola Industrial E Comercial de Nun’Álvares, Viana Do Castelo, 4900-347 Portugal; 5 Escola Superior Agrária, Instituto Politécnico de Viana Do Castelo, R. D. Mendo Afonso, 147, Ponte de Lima, Refóios, 4990-706 Portugal; 6 LABBELS –Associate Laboratory, Braga/Guimarães, Portugal

**Keywords:** *Pseudomonas syringae* pv. *actinidiae*, Bacteriophage, Bacterial canker, Kiwifruit

## Abstract

*Pseudomonas syringae* pv. *actinidiae* (Psa) is the causative agent of bacterial canker in kiwifruit (*Actinidia* spp.). Psa biovar 3 is the most prevalent and virulent, causing frequent and severe outbreaks worldwide. While current treatments have low efficacy, bacteriophages emerge as possible environmentally safe alternative biocontrol agents. In this study, bacteriophage Brt_Psa3 was isolated from the soil of a kiwifruit orchard in Portugal. Morphologically, Brt_Psa3 forms clear plaques and has a Podoviral morphotype. The bacteriophage exhibited broad lytic activity against several plant-pathogenic *Pseudomonas* strains, including Psa isolates. The isolated bacteriophage has a latent period of 100 min, a burst size of 143 particles/cell, and demonstrates stability at different temperatures and pH values found in kiwifruit orchards. In addition, Brt_Psa3 exhibited tolerance to UVA irradiation during 120 min of incubation. Brt_Psa3 belongs to the *Autographiviridae* family and *Ghunavirus* genus, based on full-genome nucleotide alignment and supported by phylogenetic analysis of structural proteins. The phage contains 51 open reading frames with no antibiotic resistance genes identified, within a genome of 40.509 base pairs. In vitro experiments with kiwifruit leaves demonstrated significant reduction of Psa levels (40%) on leaf surfaces, highlighting the bacteriophage’s therapeutic potential in managing bacterial canker in kiwifruits.

**Supplementary Information:**

The online version contains supplementary material available at 10.1007/s00253-025-13613-z.

## Keypoints

• *Isolated bacteriophage Brt_Psa3 effectively infect Psa strains*

• *Brt_Psa3 maintains infectivity under conditions similar to kiwifruit orchard environment*

• *Leaf assay confirms its biocontrol potential*

**Supplementary Information:**

The online version contains supplementary material available at 10.1007/s00253-025-13613-z.

## Introduction

Plant pathogenic *Pseudomonas* spp. have worldwide distribution and cause different diseases in diverse groups of plants. Due to their genetic diversity, ecology, and type of disease they cause, symptomatology ranges from necrotic spots, lesions, blights, and bacterial cankers to vascular infections, hyperplasias, and tissue softening (Schroth et al. 2006). The *Pseudomonas syringae* complex causes disease in more than 180 economically important crop species, including fruit trees like sweet cherry, plum (Marroni et al. 2024), and also kiwifruit (*Actinidia deliciosa)* (Pinheiro et al. 2020). *Pseudomonas syringae* pv*. actinidiae* (Psa) is a widespread pathogen, causing bacterial canker in kiwifruit plants (Figueira et al. 2020; Flores et al. 2020). Psa populations, based on genetic and phenotypic analysis, are classified into biovars (Figueira et al. 2020). Biovar 3 is the most virulent and responsible for a global pandemic of this plant disease. The characteristic symptoms of Psa infection are characterized by necrotic spots on leaves contoured by yellow halos, bacterial exudates in the trunks, and browning or darkening of vascular tissues (Ares et al. 2021).

Copper or antibiotic-based formulations are the most widely used chemicals for managing crop diseases (Flores et al. 2020). However, their excessive and uncontrolled use leads to several consequences: contamination of ecosystems, compromising food safety and human health (Cameron and Sarojini 2014; Rani et al. 2021; Tudi et al. 2021); pest resistance (Cameron and Sarojini 2014), and disruption of microbial communities that are vital for soil fertility and plant health (Tudi et al. 2021). As a result, the main focus has been placed on finding alternatives to the use of chemical control and the development of sustainable biocontrol strategies, which to date have focused mainly on bacteria/fungi-based formulations (Stark et al. 2018; Costa-Santos et al. 2021) but still present lower effectiveness than agrochemicals.

Bacteriophages represent a safer and sustainable solution for pest control due to their high specificity to target bacterial hosts (not affecting e.g., animals or plants), strong lytic activity (Sabri et al. 2021), and self-replicating and self-limiting nature exclusively depending on the presence of the host bacteria (Vu and Oh 2020). Bacteriophages are viruses that only utilize bacterial cells as a host, taking over the host metabolism in order to replicate (Rabiey et al. 2020). Like other viruses, bacteriophages are extremely ubiquitous, and there are multiple sources of bacteriophages, like soil, water, or infected plant parts, everywhere where host bacteria exist (Artawinata et al. 2023). According to the recent International Committee on Taxonomy of Viruses (ICTV) updates, the traditional families *Myoviridae*, *Siphoviridae*, and *Podoviridae* are no longer valid as well as the order *Caudovirales.* The terms Myoviral, Siphoviral, and Podoviral are now used to describe morphological traits, not taxonomic rank (Turner et al. 2023). Myoviral bacteriophages have long, rigid, contractile tails, while Siphoviral possess long, flexible, noncontractile tails. In contrast, Podoviral are characterized by short, noncontractile tails (Batinovic et al. 2019).

Bacteriophages that target plant pathogenic bacteria offer interesting opportunities for biological control. To date, several bacteriophages have been identified and studied as potential agents to combat plant-pathogenic bacteria, such as different pathovars of *P. syringae* (*P. syringae* pv. *tomato*, *P. syringae* pv. *phaseolicola*, *P. syringae* pv. *syringae*, *P. syringae* pv. *morsprunorum*, *P. syringae* pv. *porri*, and *P. syringae* pv. *actinidiae*) (Pinheiro et al. 2020; Liu et al. 2021), *Dickeya solani* (Czajkowski et al. 2014), *Ralstonia solanacearum* (Bae et al. 2012), *Erwinia amylovora* (Biosca et al. 2024), and *Xanthomonas* spp. (Stefani et al. 2021).

However, some limitations such as bacterial resistance and limited lytic spectrum should be overcome to potentiate the incorporation of phages in biocontrol strategies (Rabiey et al. 2020; Sabri et al. 2021; Luo et al. 2022; Córdova et al. 2023). Furthermore, environmental conditions such as temperature, UV radiation, and soil pH may affect the stability of the bacteriophage (Luo et al. 2022; Córdova et al. 2023), and the complexity of the interactions between the bacteriophage and its host is little studied in the environment (Córdova et al. 2023). So, the main goal of this study was to isolate and characterize a lytic bacteriophage able to kill Psa, aiming at the development of an effective biocontrol agent against bacterial canker.

## Material and methods

### Bacterial strain and growth conditions

Bacterial strains used in this study are summarized in Supplementary Table S1. All *Pseudomonas* spp. were grown in Lysogeny broth (LB) (Liofilchem, Roseto degli Abruzzi, Italy) and LB agar (LB supplemented with 1.5% (w/v) bacteriological agar [Oxoid, Thermo Scientific™, Basingstoke, England]) at 28 °C. LB soft agar used in phage purification, production, and spotting assays contained 0.3% (w/v) agar. The isolated bacteriophage Brt_Psa3 was isolated, characterized, and belongs to the Integrative Biology and Biotechnology Laboratory (iB2Lab) microorganisms’ collection, Faculty of Sciences of the University of Porto (FCUP).

### Bacteriophage isolation

Bacteriophages were isolated from different samples (leaf, flower, soil, stick, and bacterial exudate) obtained from Psa-infected kiwifruit orchards in North and Centre of Portugal, following the protocol described by Flores et al. (2020), with minor modifications. The samples were homogenized in Sodium-Magnesium (SM) buffer (100 mM NaCl, 10 mM MgSO_4_, pH 7.5), and the filtered supernatant (0.22 µm filter) was incubated with prophage-free Psa (CFBP7286) culture (OD_600_ = 0.3) overnight at 25 °C. Then, samples were centrifuged (10,000 × g for 10 min). The filtered supernatant (0.22 µm filter) was mixed with a Psa culture (grown overnight), and zones of lysis were detected using the dual Psa soft-agar lawn method (Flores et al. 2020). The bacteriophage lysate was stored at 4 °C.

### Bacteriophage host range and inhibition assays

The host range of the bacteriophage isolates was determined by the efficiency of plating (EOP) using the spot assay method against bacterial collection (Supplementary Table S1), as previously described by Witte et al. (2022). Briefly, to evaluate bacteriophage lytic activity, 10 µL of decimal serial dilutions of each bacteriophage was spotted onto the surface of double-layer agar plates containing lawns of the selected bacterial strains. The plates were incubated for 24 h at 28 °C. After the incubation period, lytic activity was classified into three categories: effectively infected (same titer as in the propagation strain (+++); if it is shifted by one level (10×, ++); if there is a shift of two levels (+) and without lytic plaques (-).

Growth inhibition curves were performed using a procedure previously described by Flores et al. (2020) with minor modifications. Psa mid-exponential cultures (OD_600_ = 0.1, ~10^7^ colony-forming units [CFU]/mL) were mixed with bacteriophage solution at a multiplicity of infection (MOI) of 0.1, 1, and 10, or SM buffer as a control. Then, 96 multi-well plates were incubated at 25 °C for 24 h in a microplate spectrophotometer. The OD_600_ was measured every 30 min. All biological assays were performed in triplicate.

### One-step growth curve

To determine bacteriophage growth parameters, a one-step growth curve was performed, as previously described by Kropinski (2018), with minor modifications. To maintain consistent conditions, the overnight bacterial culture was centrifuged (4.500× g, 5 min) and resuspended in SM buffer. Next, bacterial cultures were added to bacteriophage suspension in order to have a MOI of 0.01. The mixture was incubated at 25 °C for 10 min at 120 rpm and centrifuged at 7.000×g for 5 min to remove unabsorbed bacteriophages. The precipitate was re-suspended in SM buffer and incubated at 25 °C and 120 rpm. Samples were collected at time 0 and at time intervals of 10 min up to 200 min of incubation. The bacteriophage titer was determined using the double-layer agar method. The burst size was calculated by subtracting the initial phage titer (measured just after the adsorption phase) from the final phage titer (measured after cell lysis) and dividing the result by the number of initially infected bacterial cells (Kropinski 2018). All biological assays were performed in triplicate.

### Transmission electron microscopy (TEM)

The morphology of the bacteriophages was determined through transmission electron microscopy (TEM) (JEM-1400 microscope JEOL, Tokyo, Japan), according to Melo et al. (2014). Bacteriophage lysate (10^9^ plaque-forming units [PFU]/mL) was centrifuged (25,000× g, 4 °C, 1 h) and washed twice in tap water. Bacteriophage suspensions were deposited on copper grids with carbon-coated copper nickel grids for 2 min, stained with 2% uranyl acetate (pH 4). Individual measurements of five viral particles were taken and averaged using ImageJ (Schneider et al. 2012).

### Genome sequencing, annotation, and pairwise comparative analysis

The bacteriophage genomic DNA was extracted with a commercial kit (Norgen Biotek Corp., Thorold, ON, Canada). The concentration and quality of the extracted DNA were assessed using the nanodrop quantifications (NanoDrop One, Thermo Fisher Scientific, Waltham, MA, USA) and 1% agarose gels. Next, DNA libraries were prepared and sequenced on an Illumina NovaSeq 6000S4 flow cell with a PE150 configuration (Stabvida, Caparica, Portugal). The sequenced reads were trimmed and de novo assembled in Geneious Prime Version 2023.1 (Biomatters Ltd., Auckland, New Zealand), with BBDuk Trimmer and Geneious assembly algorithm (with medium sensitivity), respectively. The assembled genome was compared using the BlastN tool on the National Center for Biotechnology Information (NCBI) database. Genes were identified by RAST (Aziz et al. 2008) and manually annotated using BlastP, HHpred, and InterPro, using default parameters (E value of ≤ 10^−5^). Putative tRNA-encoding genes were identified using tRNAscan-SE 2.0 (Lowe and Chan 2016). Pairwise whole-genome nucleotide comparisons were visualized with Easyfig v2.2.3 (Sullivan et al. 2011).

### Phylogeny and taxonomy studies

Phylogenetic analyses were performed using amino acid sequences of structural proteins (major capsid protein and large terminal subunit) of the closest bacteriophages. Amino acid sequences were aligned using MAFFT (v7.490) (Katoh and Standley 2013) with default options, and phylogenetic trees from the alignments were constructed using the Jukes-Cantor genetic distance model and neighbor-joining tree building method using Geneious Prime (2023.1.2) (Biomatters Ltd., Auckland, New Zealand) (Kearse et al. 2012) with the bootstrap set to 100.

### Stability of the bacteriophage under thermal, ultraviolet and pH conditions

Stability of the bacteriophage under thermal, UVA radiation, and pH conditions was performed according to Flores et al. (2020), with minor modifications. Timing, temperature, pH values, and UV radiation conditions were selected based on Luo et al. (2022). To assess pH and temperature stability, the bacteriophage lysate (10^8^ PFU/mL) was suspended in 1 mL of SM buffer, at a range of pHs from 5 to 8, and then stored at 5, 15, 25, 35, and 45 °C for 1 week, in order to evaluate the combined effect of pH and temperatures. To evaluate bacteriophage survival under solar radiation, bacteriophage suspensions in SM buffer were exposed to UV radiation with wavelengths between 315 and 380 nm (PL-L 36W/09/4P UV-A, Philips, Bielsko‑Biała, Piła, Kętrzyn). Samples were taken at 0, 30, 60, 120, and 180 min. Following incubation under each condition, serial dilutions of the treated bacteriophage samples were titrated using the corresponding Psa host in a double-layer agar assay. All experiments were conducted in biological triplicate.

### In vitro experiments of bacteriophage treatment of Psa infected kiwifruit leaves fragments

In vitro assay was used to assess the impact of bacteriophages on the Psa population on the surface of kiwifruit leaves, according to Pinheiro et al. (2020) with some modifications. In addition, the impact of bacteriophages on the damage caused by Psa on the leaf squares was conducted following the protocol outlined by Flores et al. (2020). According to the percentage of the leaf surface fragments showing necrosis, a Disease Index (DI) scale (0–4) recommended by Prencipe et al. (2018) was used to quantify the Psa damage, where 0 means 1–4 percentage of the leaf area necrosed; 1: 5–10%, 2: 11–30%, 3: 31–60%, and 4: >60% of leaf necrosis. In this assay, four conditions were evaluated: condition 1: only Psa was applied; condition 2: only bacteriophage was applied; condition 3: bacteriophage at MOI 10 was applied after 2 h of Psa infection to allow the bacterial suspension to dry on the leaf surface; and condition 4: control, where the same volume of deionized H_2_O was applied.

Leaf squares (3 × 3 cm) were obtained from two-year kiwifruit plants (*Actinidia chinensis* var. *deliciosa* “Hayward”) maintained under greenhouse conditions. The fragments’ surface was disinfected with H_2_O_2_ at 3% (v/v). Next, kiwifruit fragments were infected with 100 µL of Psa (CFBP7286) at ~ 10^7^ CFU/mL. After 2 h, 100 µL of bacteriophage suspension (MOI = 10) was spread on the leaf surface. Samples were placed in Petri plates (90 mm) containing 10 mL of deionized H_2_O (to prevent dehydration) and incubated at 25 °C. At each time point (0, 24, 48, and 72 h), leaf samples were placed in 10 mL PBS and incubated for 30 min at 130 rpm. The bacteria and bacteriophage concentrations were determined using the drop-plate method. This experiment was performed using leaves from different plants, and three independent experiments were performed for each condition.

### Accession number

The genomic sequence of the *Pseudomonas* phage Brt_Psa3 was deposited in the NCBI GenBank database with the accession number PQ453564.

### Statistical analysis

The statistical analysis was conducted using GraphPad™ version 9.5.0 (GraphPad Software, San Diego, CA, USA). A two-way ANOVA followed by Tukey’s multiple comparisons test was employed to assess the time-dependent effects of temperature and pH. The stability under UVA radiation and the in vitro bacteriophage treatment experiments were evaluated using one-way ANOVA with Tukey’s test of multiple comparisons. All experiments were performed in both biological and technical triplicates, and a *p*-value of less than 0.05 was deemed statistically significant.

## Results

### Bacteriophage isolation and morphology

In order to characterize Psa bacteriophage population, different samples (leaf, flower, soil, stick, and bacterial exudate) were collected from eight Psa-infected kiwifruit orchards. In total, 41 bacteriophages were isolated (data not shown), among which only one was virulent (Brt_Psa3), while the others were temperate, indicating a very low rate of lytic bacteriophage isolation and the prevalence of temperate bacteriophages. Therefore, the main focus of this study was on lytic bacteriophages.

**Fig. 1:**
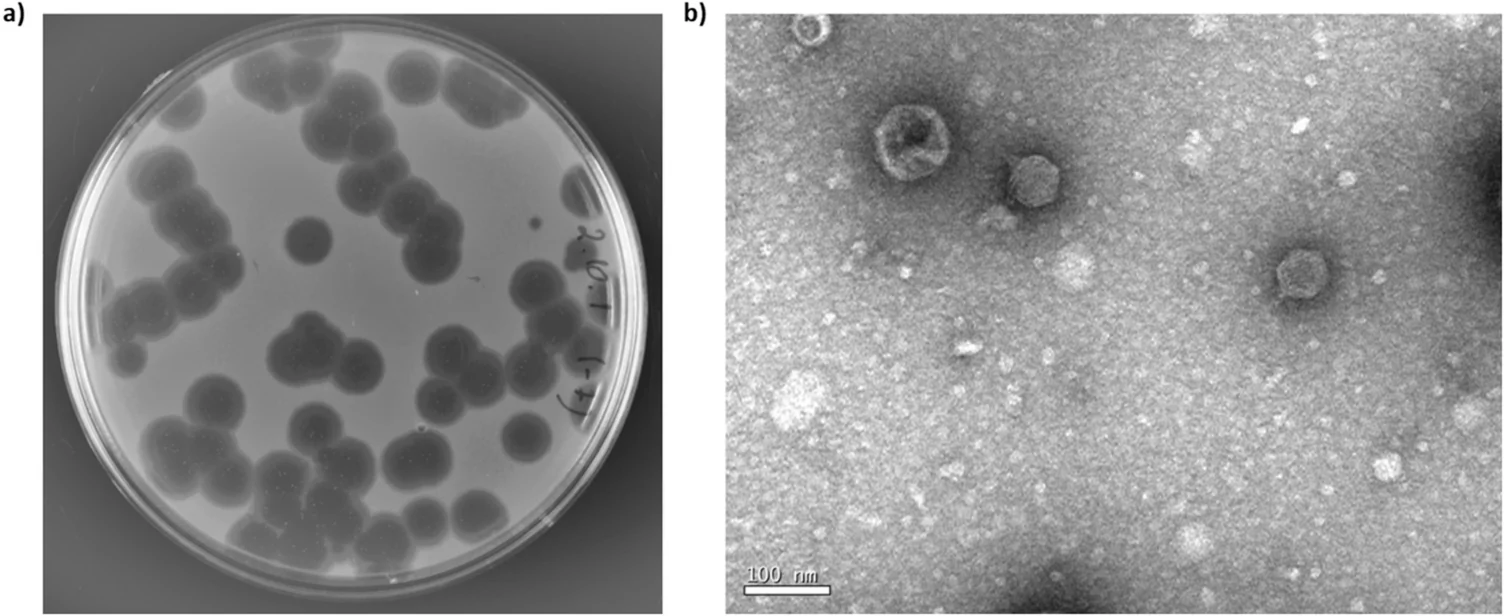
Bacteriophage Brt_Psa3 characteristics. **a**) Phage plaques formed on a lawn of the bacterial host (CFBP7286). **b**) Virion morphology observation of Brt_Psa3 under TEM

Bacteriophage Brt_Psa3 was isolated from a soil sample collected in a Psa-infected kiwifruit orchard in Brito, Portugal. Brt_Psa3 forms clear plaques approximately 6–7 mm in diameter with smooth borders on a bacterial lawn of the host strain CFBP7286 (Fig. 1a). The TEM analysis showed that the isolated bacteriophage has typical Podoviral morphology, exhibiting an isometric capsid of 59 ± 1.0 nm in diameter and a 10 ± 1.0 nm short tail (Fig. 1b).

### Bacteriophage host range and genomic characterization

The host range of Brt_Psa3 was determined by a spot test of serial dilutions of the phage on lawns of 50 strains of *P. syringae* pv. *actinidiae* biovar 3, among which 43 strains were isolated from infected kiwifruit orchards in North and Central Portugal. The results showed that only 25 strains were effectively infected, among which were 22 Psa biovar 3 strains, *Pseudomonas cerasi*, and *Pseudomonas viridiflava* (Supplementary Table S2).

### Genomic and phylogenetic characterization of isolated bacteriophage

**Fig. 2:**
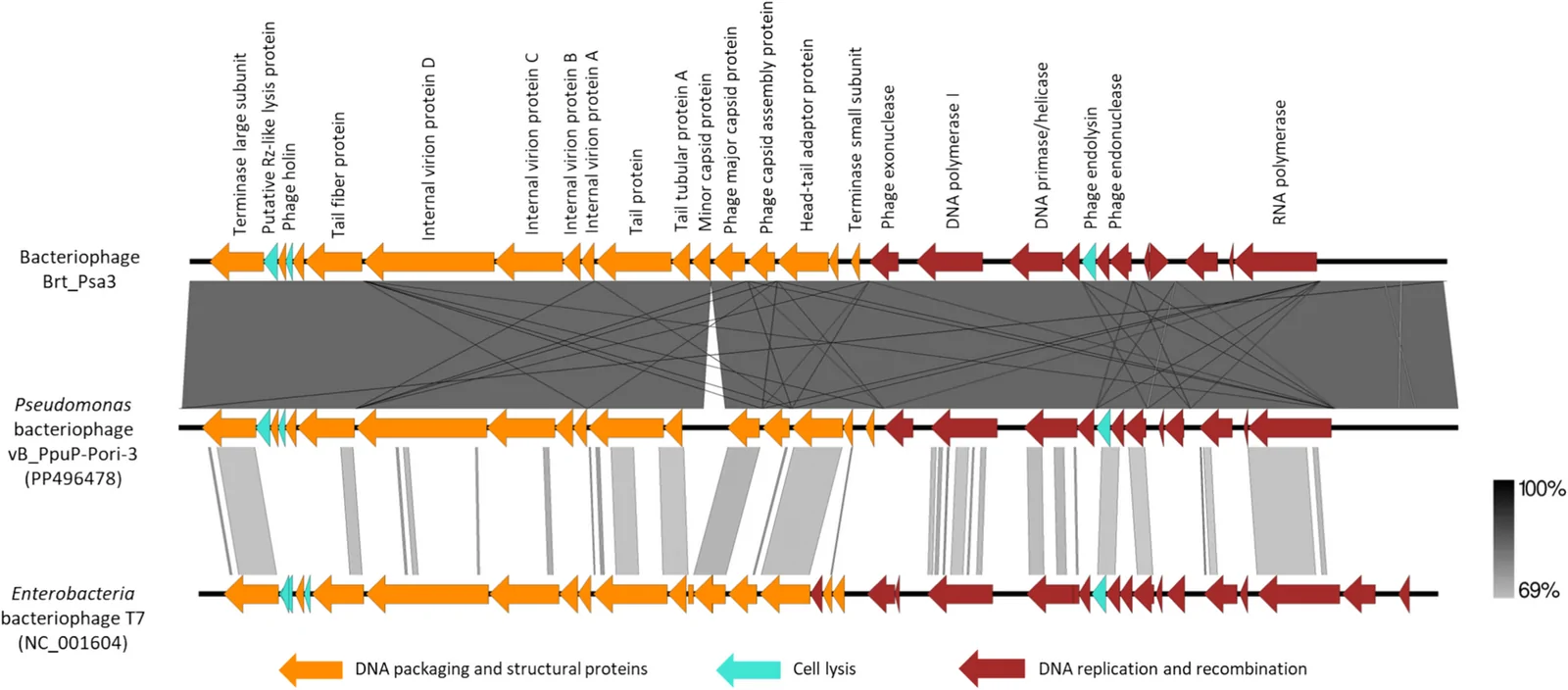
Comparison of the genomes of bacteriophage Brt_Psa3, *Pseudomonas* bacteriophage vB_PpuP-Pori-3 (PP496478), and *Enterobacteria* bacteriophage T7 (NC_001604). Colored arrows represent the coding sequences (CDS) across the full genomic length, with the arrow direction indicating the transcription direction of each CDS. Homologous regions are depicted by slanted gray bars, with the intensity of the gray color reflecting the degree of sequence similarity

The linear double-stranded DNA genome of bacteriophage Brt_Psa3 contains 40.509 base pairs (bp) with a GC content of 56.8%, which is slightly lower than that of its Psa host strain CFBP7286 (58.5%). The analysis of the Brt_Psa3 genome revealed 51 open reading frames (ORFs) oriented in the reverse direction. Among the 51 open reading frames (ORFs), 17 gene products have unknown functions, and 34 have predicted functions (Supplementary Table S3). ORFs encoding proteins with known functions were classified into three groups: those associated with DNA replication and recombination, DNA packaging and structural proteins, and cell lysis (Fig. 2).

The bacteriophage genome contains no tRNA genes, and no genes coding for antibiotic resistance were detected. No integrases were found on the Brt_Psa3 genome, and the presence of enzymes responsible for cell lysis, like ORF27 endolysin, ORF42 holin, and ORF25 putative Rz-like lysis protein was detected.

Interestingly, whole-genome sequence analysis obtained using the BLASTn alignment method indicated that the isolated bacteriophage is closely related to *Pseudomonas* bacteriophage vB_PpuP-Pori-3 (PP496478.1, with 99.14% sequence identity and 100% of query coverage) and *Pseudomonas* bacteriophage shl2 (NC_048200.1, with 83.97% sequence identity and 85% of query coverage), which have different isolation hosts, *Pseudomonas *putida - KT2440 and Paw85, and *P. syringae* pv. *tomato* (DC3000), respectively. Thus, the isolated phage was assigned as a member of the class *Caudoviricetes* and the *Autographiviridae* family. The difference is evident in the coding sequences (CDS) for the tail proteins, and single nucleotide polymorphisms (SNPs) are also observed in other regions, including other structural, DNA replication, and recombination proteins.

The amino acid sequences of major capsid protein and terminase large subunit were used to study their taxonomic relationship. To construct the trees, we used homologous proteins detected using the BLASTp tool. A phylogenetic analysis using alignments of these sequences clustered bacteriophage Brt_Psa3 with other *Autographiviridae Ghunavirus* bacteriophages (Supplementary Fig. S1).

### Characterization of bacteriophage lytic activity

**Fig. 3:**
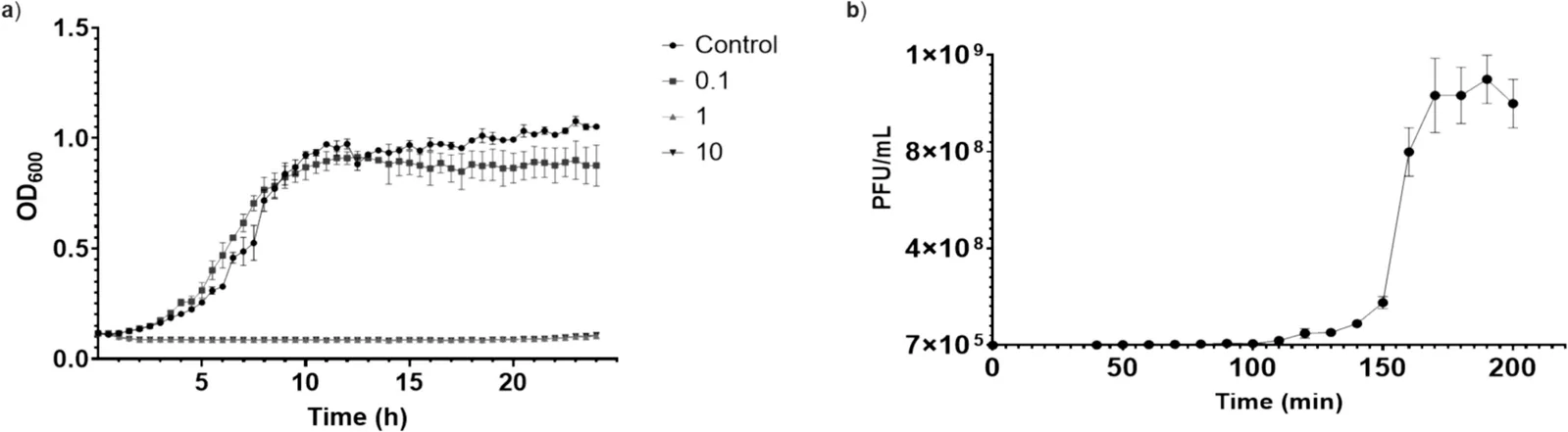
Lytic activity of bacteriophage Brt_Psa3. **a**) Inhibition curve at an MOI 0.1, 1 and 10 on the Psa host strain (CFBP7286). The control curve corresponds to the bacterial culture without the addition of bacteriophage. The OD_600_ of the bacterial culture was measured at each 15 min during the incubation period. **b**) One-step growth curve of bacteriophage Brt_Psa3. Error bars represent the standard deviation, and the assay was repeated three times

The lytic activity of bacteriophage Brt_Psa3 was assessed through an inhibition assay using the Psa type strain (CFBP7286). The results show that at multiplicities of infection (MOIs) of 1 and 10, the bacteriophage was able to reduce the optical density (OD) of the bacterial cultures during the first 2 h. However, from 19 to 24 h, the OD of the cultures gradually increased, indicating partial regrowth of the bacterial population. At a MOI of 0.1, the reduction in OD was not significantly different from the control (*p* < 0.05) (Fig. 3a). The infection kinetics of the bacteriophage was evaluated by one-step growth curve. The latent period was approximately 100 min, a rise period of 70 min, and a burst size of 143 bacteriophage particles per infected host cell (Fig. 3b).

### Stability of the bacteriophage under various thermal, ultraviolet, and pH conditions

**Fig. 4:**
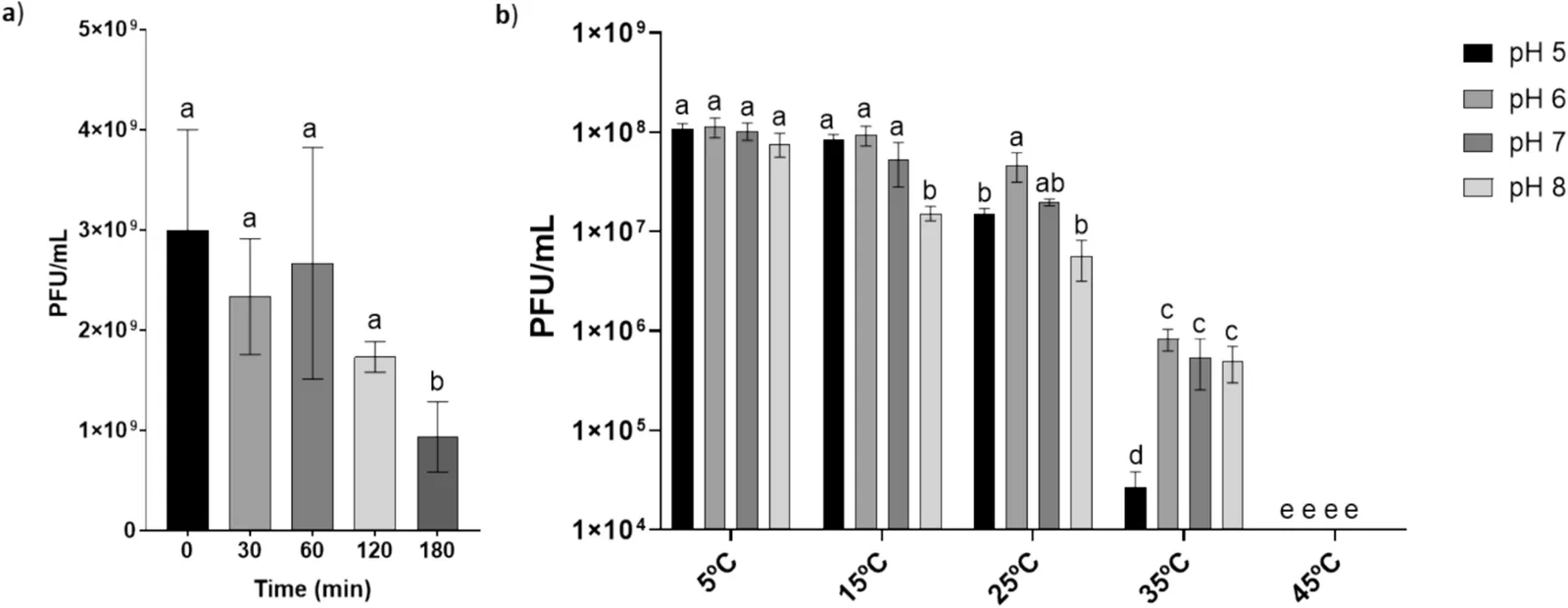
Stability of the bacteriophage Brt_Psa3 under **a**) UVA radiation (315 to 380 nm) up to 180 min and **b**) different temperatures (5, 15, 25, 35 and 45 °C) and pH (5, 6, 7 and 8) combination after 1 week of incubation. The assay was repeated three times and analyzed in GraphPad with two-way ANOVA. Standard deviation (sd) is shown for each bar and distinct letters at the tops of the bars indicate significant variations in bacteriophage titer (PFU/mL)

First, Brt_Psa3 infectivity was evaluated under UVA radiation. It was observed that during 180 min of incubation, the bacteriophage titer gradually decreased, demonstrating a significant difference at the last time point (Fig. 4a). Next, in order to evaluate Brt_Psa3 stability under environmental conditions found in kiwifruit orchards, the bacteriophage was exposed to temperature and pH combinations, considering 25 °C and pH 7 as reference parameters. Brt_Psa3 titer remained high (approximately 10^8^ PFU/mL) with no statistically significant differences observed between treatments, particularly at low temperatures (5 and 15 °C) across all tested pH values. At 25 °C, a significant decrease in bacteriophage titer was observed at pH 5 and pH 8. When the temperature was increased to 35 °C, the titer declined significantly across all pH levels. The lowest titer was observed at pH 5, which was significantly lower than at pH 6, 7, and 8. At 45 °C, the bacteriophage was completely inactivated.

### In vitro experiments of bacteriophage treatment of Psa infected kiwifruit leaves squares

**Fig. 5:**
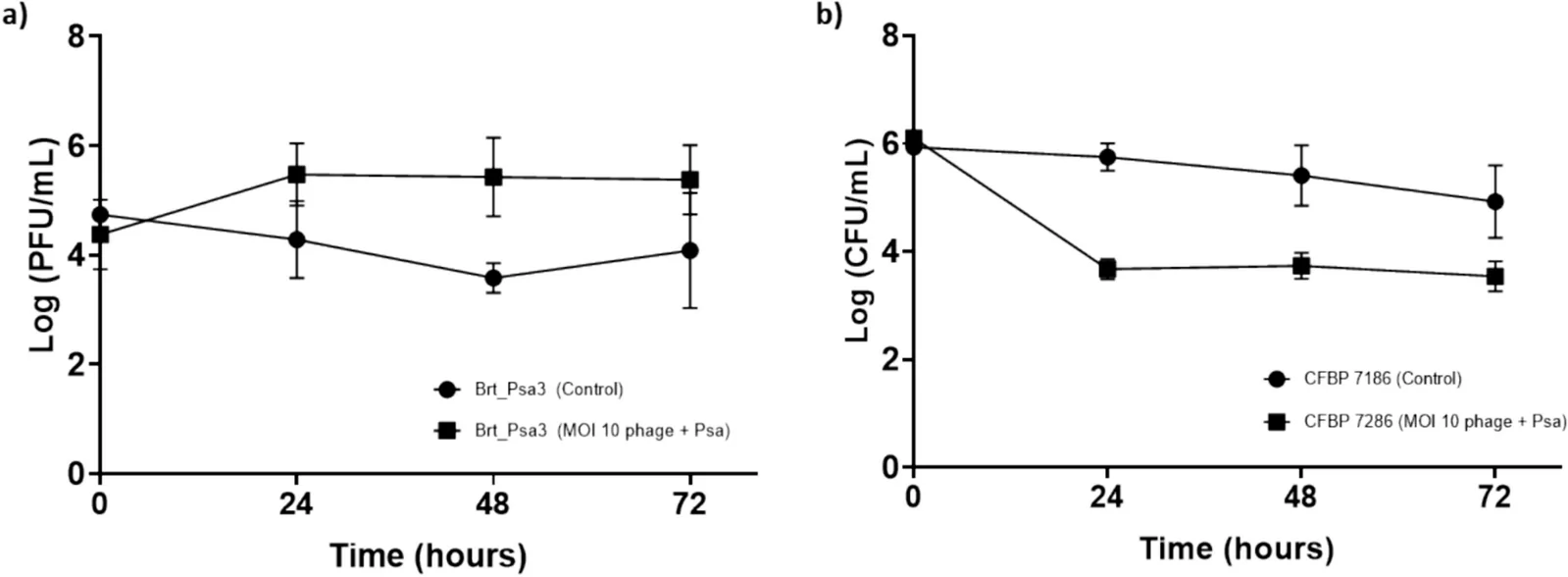
In vitro infection of Psa host (CFBP 7286) by bacteriophage Brt_Psa3 at MOI 10. The **a**) bacteriophage and **b**) bacteria concentration was analyzed at time point 0, 24, 48 and 72 h post inoculation. Values indicate the average of three separate assays; error bars show the standard deviation

Conventional in vitro plaque assays, while useful for initial characterization, do not replicate the complex and dynamic conditions encountered on plant surfaces. Therefore, to obtain more realistic and reliable data on phage performance, in planta assays were conducted to evaluate the effectiveness of bacteriophage Brt_Psa3 under biologically relevant conditions. The Psa population on leaf surface (control) slightly decreases by 5.93 to 4.92 log CFU/mL (*p* < 0.05) after 72 h of incubation (Fig. 5b). A similar trend was observed for the bacteriophage Brt_Psa3 control (without the host) (Fig. 5a), where the bacteriophage titer decreased from 4.74 to 4.08 log CFU/mL (*p* < 0.05) in a 72 h period (Fig. 5a). In contrast, when the bacteriophage was applied to bacterial-infected leaves, a significant reduction in Psa levels was observed (from 6.10 to 3.67 log CFU/mL (*p* > 0.05)) (Fig. 5b) and an increase in bacteriophage titer (from between 4.38 to 5.47 log PFU/mL (*p* > 0.05)) during the first 24 h period, suggesting that the bacteriophage treatment exerted a rapid bactericidal effect (Fig. 5a).

The continued reduction in Psa levels after a 72-h period further supports the biocontrol potential of bacteriophages. Interestingly, no significant variations in the bacteriophage titer were observed between 24 and 72-h periods. This could suggest that the bacteriophages may have reached an equilibrium state, where their replication rate balances with the decrease in available bacterial cells. Such a trend may also indicate a limit to the bacteriophage’s impact after the initial burst, which might be due to the reduced bacterial load or the emergence of resistance mechanisms.

**Fig. 6:**
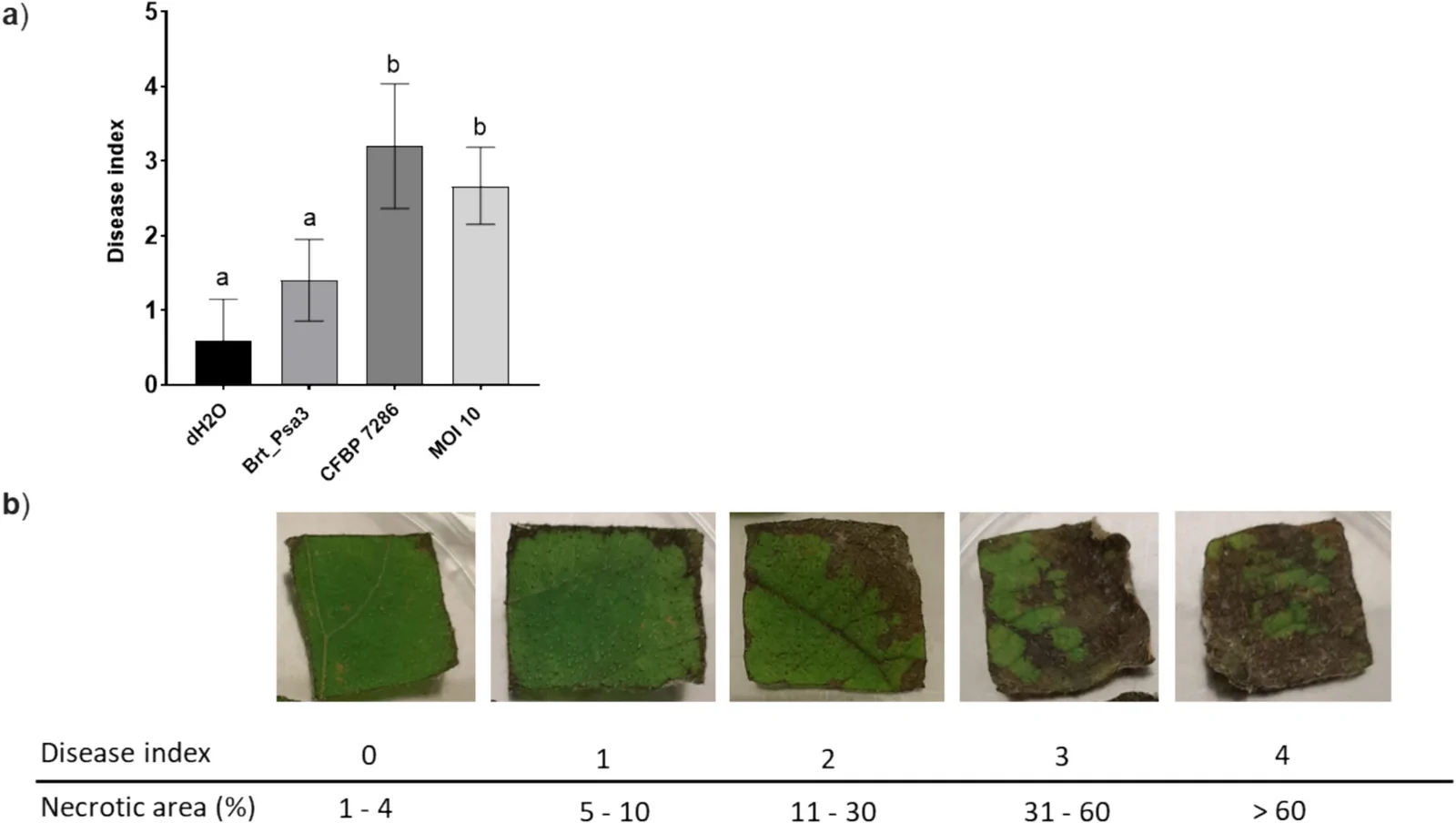
The **a**) disease index in kiwifruit leaf squares 72-h post-infection with Psa (CFBP7286) and **b**) the visual percentage of the leaf area necrosed (0–4) employed to analyze the experiment. Leaf squares treat with dH_2_O were used as the control. The experiment was done using three kiwifruit squares and analyzed in GraphPad™ by a two-way ANOVA. Different letters at the top of the bars means significant differences in disease index among treated and untreated squares

At the same time, we evaluated the effect of bacteriophage treatment on symptom development in kiwifruit leaves. Previous results indicated that bacteriophage Brt_Psa3 was able to reduce Psa population on the leaf surface. However, a minimal decrease in the disease index was observed, with no significant difference between the bacteriophage-treated (average disease index of 2.7) and the leaves only infected with Psa (average disease index of 3.2) (*p* > 0.05) (Fig. 6a). Interestingly, an unexpected increase in the disease index was observed between non-treated leaf squares (average disease index of 0.6) and those where the bacteriophage was applied (average disease index of 1.4) (*p* < 0.05) (Fig. 6a). This result suggests a possible phytotoxic effect or stress response triggered by the bacteriophage in the absence of the bacterial host, needing further investigation.

## Discussion

The most critical prerequisite of successful bacteriophage therapy is the presence of appropriate lytic bacteriophage that can recognize and effectively lyse the host bacteria (Batinovic et al. 2019; Nawaz et al. 2023). So, without the large and diverse bacteriophage source bank, the practical bacteriophage application would be limited and less effective (Frampton et al. 2014; Buttimer et al. 2017). Therefore, selected bacteriophages for the phage biocontrol must possess characteristics such as high specificity, strict virulence, environmental persistence, and genomic safety (Buttimer et al. 2017; Fong et al. 2021; Nawaz et al. 2023). Numerous sources, including soil (Martino et al. 2021), water (Di Lallo et al. 2014; Ni et al. 2021), and plants (Silva et al. 2023), could be potential reservoirs of bacteriophages. However, even in trees that exhibit symptoms, it is difficult to isolate virulent bacteriophages from the aerial components. The primary bacteriophage reservoir may be the soil, where the bacteriophages can multiply and are protected from the environmental conditions.

High prevalence of temperate bacteriophages was observed during bacteriophage isolation. The high number of these bacteriophages may be related to the presence of Psa or other closely related bacteria in the samples and spontaneous prophage induction. Since we used different samples from Psa-infected kiwifruit orchards, other Psa strains could proliferate, and spontaneous prophage induction may potentially influence the observed temperate bacteriophage prevalence. The same phenomenon was observed by Dougherty et al. (2023), which detected spontaneous prophage induction on *Pseudomonas* strains. Similar temperate phages were also isolated by Di Lallo et al. (2014) and Rabiey et al. (2020) from Psa-infected leaves and soil, respectively. In fact, temperate bacteriophages are protected from environmental conditions when they integrate and replicate with their host, contributing to bacterial fitness and pathogenicity (Greenrod et al. 2022). At the same time, these conditions (e.g., temperature and radiation) (Yue et al. 2012) and agrochemical application (e.g., copper) (Zhang et al. 2022) could influence temperate bacteriophage abundance by activating the lytic cycle. So, only strictly lytic bacteriophages with important traits like high specificity to target bacteria, strong virulence, environmental stability, and absence of virulence genes are selected for phage therapy (Choudhary et al. 2025).

One lytic bacteriophage was successfully isolated, namely Brt_Psa3 against *P. syringae* pv. *actinidiae* (Psa). Brt_Psa3 genome does not encode bacterial virulence factors, antibiotic resistance, and lysogenic-related genes, indicating its genomic safety. Its genomic characteristics, phylogenetic relationships, and functional similarities clustered the isolated bacteriophage with other *Ghunavirus* phages. Genomic studies of bacteriophages with Podoviral morphology have indicated that Psa bacteriophages identified in Chile (CHF1, 7, 19, and 21) (Flores et al. 2020), New Zealand (phiPsa17) (Frampton et al. 2014), Italy (Di Lallo et al. 2014), and South Korea (PPPL-1) (Park et al. 2018) are closely related and were grouped in the same cluster (Luo et al. 2022). The comparison of Psa bacteriophages across different countries provides insight into the global distribution of these viruses (Liu et al. 2021; Luo et al. 2022). This observation may reflect the pandemic spread of Psa, indicating a potential association between the bacteriophage and host.

Bacteriophage demonstrated a broad strict lytic spectrum compared to other bacteriophages, successfully infecting 35% of bacterial strains used in this study. Interestingly, *P. cerasi* and *P. viridiflava* were also infected with high efficiency; both strains were isolated from Psa infected leaves. Co-occurrence of *P. viridiflava* and Psa is well documented (Purahong et al. 2018), but *P. cerasi* and Psa are limited. Certain bacteriophages can not only effectively target multiple pathovars of *Pseudomonas syringae*, but could also infect other *Pseudomonas* species, such as *P. viridiflava* and *Pseudomonas fluorescens* (Frampton et al. 2014). Supporting this, other studies have shown that bacteriophages targeting *P. syringae* pv. *actinidiae* (Psa) can infect various other pathovars, including *P. syringae* pv. *morsprunorum*, *Pseudomonas syringae* pv. *tabaci*, *P. syringae* pv. *tomato*, *P. syringae* pv. *phaseolicola*, *Pseudomonas syringae* pv. *theae*, *Pseudomonas avellanae*, *P. syringae* pv. *syringae* as well as *Pseudomonas savastanoi* pv. *fraxinii* (Di Lallo et al. 2014; Frampton et al. 2014; Park et al. 2018; Pinheiro et al. 2019; Martino et al. 2021; Silva et al. 2023). However, it is important to note that not all bacteriophages have a similar host range. For instance, PPPL-1 showed no activity against *Pseudomonas* pathovars such as *P. syringae* pv. *tomato*, *P. syringae* pv. *tabaci*, *P. syringae* pv. *actinidiae* (SYS3), and other bacterial species such as *P. fluorescens* (Park et al. 2018). This susceptibility suggests that Psa bacteriophages may recognize receptors shared across the *P. syringae* species complex which are located at the lipopolysaccharide (LPS) layer, flagella, pili, or outer membrane proteins, with the LPS layer being the most commonly targeted site for bacteriophage adsorption (Fong et al. 2021; Martino et al. 2021).

What is currently observed is that several pathogenic agents can coexist and have more than one host plant. Bacteriophage lytic spectrum can vary significantly between strains, even within the same species. While some have a broad spectrum of action, others are highly specific, which limits their application. Thus, the use of bacteriophage cocktails with bacteriophages with varied lytic spectra may be a viable strategy to ensure effective control of *Pseudomonas* complex. The narrow host range of individual Psa bacteriophages means they have minimal to no impact on non-pathogenic species, like beneficial microflora. Additionally, Brt_Psa3 demonstrated strong inhibitory activity, restricting bacterial growth at MOI 1. Other bacteriophages (CHF1, CHF7, CHF19, and CHF21) have been shown to effectively reduce the optical density of bacterial cultures, even at a multiplicity of infection (MOI) of 0.1, but this effect was only evaluated after 18 h of incubation (Flores et al. 2020). For instance, Di Lallo et al. (2014) monitored the activity of the φPSA2 bacteriophage for up to 24 h and observed that, although absorbance initially decreased drastically at an MOI of 0.01, bacterial regrowth occurred after 20 h of incubation. A similar trend was observed by Park et al. (2018), where absorbance gradually decreased up to 12 h at the same MOI, but then slowly increased over the following 80 h. This tendency can indicate the development of resistance to the bacteriophage. As bacteria adapt, they can develop mechanisms like changing cell surface receptors, inhibiting phage adsorption, or interfering with phage replication and lysis processes, which can result in the regrowth of the bacterial population (Luo et al. 2022; Córdova et al. 2023). This phenomenon highlights the need for robust bacteriophage selection for cocktail formulation to bypass resistance development (Frampton et al. 2014; Buttimer et al. 2017; Córdova et al. 2023).

The latent period of bacteriophage Brt_Psa3 was longer (100 min) compared with other Psa-infecting phages like φPSA2 (15 min) (Di Lallo et al. 2014) and φXWY0026 (15 min) (Yin et al. 2019). In fact, the latent period of a lytic bacteriophage can vary widely depending on a combination of different factors, including the complexity of the bacteriophage’s life cycle, the growth rate and physiological state of the host bacterium, the specific interactions between bacteriophage and host, and physiological conditions like osmolarity, temperature, pH, and electrolyte requirements (Nabergoj et al. 2018). Particularly, Pinheiro et al. (2020) observed that the dynamics of bacteriophage-host replication change according to host bacteria. The latent period between two hosts, *P. syringae* pv. *syringae* (DSM 21482) and *P. syringae* pv. *actinidiae* (CRA-FRU 12.54), did not change, but the burst size varied: 60 ± 1 and 148 ± 1 PFU/host cell, respectively. In the case of the burst size, according to other Psa bacteriophages φPSA2 (92 PFU/host cell) (Di Lallo et al. 2014) and φXWY0026 (170 PFU/host cell) (Yin et al. 2019), Brt_Psa3 (143 PFU/host cell) was effectively reproduced in the host bacterial cells. A high burst size means more virions are available to infect other cells, which is crucial for the success of biocontrol, especially in agricultural settings. Thus, selecting bacteriophages with optimal lytic activity and burst size is vital for effective disease control (Luo et al. 2022).

Another requirement for successful bacteriophage therapy is stability under different environmental conditions, such as temperature, pH, and UV radiation (Buttimer et al. 2017; Luo et al. 2022; Vila et al. 2024). First, Brt_Psa3 infectivity was evaluated under UVA radiation. The bacteriophage was stable during the first 2 h, with a decrease in titer observed after three hours of incubation. For instance, studies by Yu et al. (2016) and Park et al. (2018) indicated that bacteriophage viability decreased after just 1 h of UVA exposure, with even more observed under UVB. The impact of solar radiation has also been investigated; Flores et al. (2020) found no significant changes in bacteriophage titer after 30 to 60 min of sunlight exposure (UV level 8). In contrast, Pinheiro et al. (2019) reported a reduction in titer after six hours of exposure to solar radiation (83.3 kWh/m^2^/day). This reduction of bacteriophage titer may result from the damage of bacteriophage capsid proteins and nucleic acids (Pinheiro et al. 2019; Luo et al. 2022). Furthermore, in order to simulate orchard conditions, Brt_Psa3 stability was investigated, combining both temperature (e.g., winter to summer) and pH conditions (e.g., soil and internal vascular tissues). The results demonstrated that both pH and temperature affect Brt_Psa3 stability. The bacteriophage remained stable at lower temperatures, maintaining high titer independent of pH values. However, as temperature increased, its stability declined, particularly under acidic (pH 5) and alkaline (pH 8) conditions. This was also observed at 35 °C at pH 5. These results indicate that acidic environments exacerbate the negative effects of elevated temperatures on bacteriophage stability. So, to minimize the effects of temperature and UV radiation, it is necessary to adopt some precautions after bacteriophage therapy, like selecting appropriate timings for applications (end of the day) or environmental conditions (after periods without rain) (Flores et al. 2020; Vu and Oh 2020; Córdova et al. 2023). In general, Psa bacteriophages can maintain their activity across a wide pH range of 2 to 12, with an optimal pH between 6 and 8 (Luo et al. 2022). For instance, φPSA2 remained stable between pH 4 and 9 after an hour of incubation (Di Lallo et al. 2014), while PPPL-1 was active across an even broader range, from pH 3 to 11 (Park et al. 2018). Similarly, φXWY0026 retained its activity from pH 2 to 12 (Yin et al. 2019). In contrast, Flores et al. (2020) observed variation in tolerance to acidic conditions at 4 and 5 pH between phages with Podoviral morphology; while CHF19 and CHF21 were significantly affected, other phages such as CHF1 and CHF7 maintained stability under these conditions. In terms of temperature, some bacteriophages are active at temperatures up to 40 °C (Di Lallo et al. 2014; Park et al. 2018; Yin et al. 2019; Flores et al. 2020), while others lose their activity at 37 °C (Flores et al. 2020). These findings demonstrate that bacteriophage stability can vary not only between studies but also between isolates under the same conditions. High temperatures not only impact bacteriophage stability, but they can impact host bacteria as well (Rombouts et al. 2016).

The inhibition activity of bacteriophage is usually higher in vitro than in planta*.* The decrease of bacterial concentration and bacteriophage titer under control conditions indicates that the surface of the leaves is a challenging environment for bacterial survival, as described by Batinovic et al. (2019). Notably, Donati et al. (2020) highlighted that, similar to other *Pseudomonas* species, *P. syringae* pv. *actinidiae* (Psa) can invade various plant organs, including leaves, by entering through natural openings such as stomata and trichomes, using polar flagella to navigate and explore leaf surfaces (Pinheiro et al. 2020). In the case of bacteriophages, the absence of the bacterial host leads to a decrease in the bacteriophage population over time, highlighting the importance of the bacterial host in maintaining phage concentration. It was observed that after treatment under field conditions, bacteriophage populations rapidly decline and are almost eliminated within 36–48 h after application (Vu and Oh 2020).

The bacteriophage treatment significantly reduced bacterial population, and consequently, the increase in the bacteriophage titer was observed during the first 24-h, which has also been shown by Pinheiro et al. 2020. Interestingly, while bacterial concentration modestly declined over 72 h, the bacteriophage titer remained stable. This suggests a possible equilibrium state, where phage replication is balanced by the decreasing availability of host cells, indicating a potential limit to phage activity after the initial infection burst, possibly due to reduced bacterial load or emerging resistance. In terms of disease symptomatology, the decrease in bacterial concentration was not proportional to a disease index. The minimal decrease in disease index was observed between bacteriophage-treated and untreated Psa-infected leaves. Bacteriophages might not be able to reverse the damage already inflicted, and their impact could be more visible in preventing further damage rather than healing existing lesions. Particularly, Song et al. (2021) demonstrated that bacteriophage therapy applied after 2 h post-infection was ineffective in plant disease progression. In contrast, Flores et al. (2020) verified that after bacteriophage cocktail application, there was inhibition of bacterial growth and reduction of disease development. The main difference between the two studies is the use of a bacteriophage cocktail, which increased the efficiency of the treatment, not forgetting other factors that could also influence the effectiveness, such as the timing of application, bacterial strains, and host plant.

The membrane components, flagella, endotoxins, and enzymes resulting from the bacteriophage lytic cycle (Binte Mohamed Yakob Adil et al. 2024) may trigger the plant’s immune system, leading to unexpected plant responses or stress. A slight increase in the disease index was observed between leaves treated only with bacteriophage and untreated control. Although it is unclear whether phytotoxicity results from cellular debris, lytic phage, or a combination of both—representing a limitation to phage therapy—phages are especially effective against bacteria, but some studies indicate the possible interaction of phage proteins with the plant's immune system. Recently, Skliros et al. (2023) observed the expression of genes associated with plant defense mechanisms at the early stages of infection after foliar bacteriophage application. However, it is not clear whether this result is due to viral particles or cellular debris present in the phage lysate. Subsequent purification of viral particles using methods like Triton X-100, CsCl ultracentrifugation, or Pierce™ High-Capacity Endotoxin Removal Resin spin column may help better understand and test these hypotheses (Binte Mohamed Yakob Adil et al. 2024). In fact, Psa encodes several type III secretion system (T3SS) effectors like AvrE1d, HopD2a, and HopR1b that directly affect host immunity, undermining both pattern-triggered immunity (PTI) and effector-triggered immunity (ETI) (Jayaraman et al. 2023). In addition, it was observed that Psa also produces exopolysaccharides (EPSs), demonstrating significant phytotoxicity on both lemon fruit and tobacco leaves (Cimmino et al. 2017). Therefore, the impact of bacteriophage activity on plant health requires further investigation to better understand the complex interactions between bacteriophages, plants, and bacterial pathogens.

## Conclusion

This study highlights the importance of soil as a valuable source of bacteriophage. Bacteriophage Brt_Psa3 was successfully isolated and characterized. Genomic analysis confirmed the absence of bacterial virulence genes, reinforcing its safety for application. Besides its low inhibition activity on the leaf surface, the isolated phage demonstrated high environmental stability and a broad lytic spectrum. We believe that combining Brt_Psa3 with other phages in a cocktail could enhance its effectiveness. So, its biocontrol potential in greenhouse and field conditions, alone and in combination with other bacteriophages, as well as the plant response to phage biocontrol still needs further investigation.

## Supplementary Information

Below is the link to the electronic supplementary material.

ESM1 (DOCX 881 KB)

## Data Availability

The authors declared that the data that supports your results and analyses are included in this article and its supplementary information. The phage genome was annotated and submitted to the NCBI GenBank database with the accession code PQ453564.
