# Supplementary material for: Lytic properties and genomic analysis of bacteriophage Brt_Psa3, targeting Pseudomonas syringae pv. actinidiae
Source: Appl Microbiol Biotechnol. 2025 Oct 11;109(1):220. doi: 10.1007/s00253-025-13613-z (PMC12515123; doi:10.1007/s00253-025-13613-z)
Supplement: Supplementary file 1 — (DOCX 881 KB) [file 253_2025_13613_MOESM1_ESM.docx]

**Applied Microbiology and Biotechnology**

These supplementary materials belong to:

Lytic properties and genomic analysis of bacteriophage Brt_Psa3, targeting *Pseudomonas syringae* pv. *actinidiae*

Emil Gimranov^1,2,3,4*^, Hugo Oliveira^1,6^, Conceição Santos^2,3^, Luísa Moura^4,5^, Joana Azeredo^1,6^

^1^Centre of Biological Engineering, University of Minho, Campus de Gualtar Braga, 4710-057 Braga, Portugal (hugooliveira@ceb.uminho.pt (O.H.); jazeredo@deb.uminho.pt (A. J.).

^2^Biology Department, Faculty of Science, University of Porto (FCUP), 4169-007 Porto, Portugal (up201804355@edu.fc.up.pt (E.G.); csantos@fc.up.pt (C.S.)

^3^LAQV-REQUIMTE, Biology Department, Faculty of Science (FCUP), University of Porto, 4169-007 Porto, Portugal

^4^CISAS—Center for Research and Development in Agrifood Systems and Sustainability, Instituto Politécnico de Viana do Castelo, Rua Escola Industrial e Comercial de Nun’Álvares, 4900-347 Viana do Castelo, Portugal

^5^Escola Superior Agrária, Instituto Politécnico de Viana do Castelo, R. D. Mendo Afonso, 147, Refóios, 4990-706 Ponte de Lima, Portugal

^6^LABBELS –Associate Laboratory, Braga/Guimarães, Portugal

*Corresponding author: Emil Gimranov (up201804355@edu.fc.up.pt)

**Supplementary Table S1.** *–* Bacterial strains used in this study. CFBP: Collection Française de Bactéries associées aux Plantes, France; ICMP: International Collection of Microorganisms from Plants, New Zealand. Bacterial strains (a) belong to the Integrative Biology and Biotechnology Laboratory (iB_2_Lab) microorganisms’ collection, Faculty of Sciences of the University of Porto (FCUP), (b) were provided by Professor Fernando Tavares, Microbial Diversity and Evolution group, Faculty of Sciences of the University of Porto (FCUP), and (c) by Dr. Joël Pothier, Microbial Genomics group at the ZHAW School of Life Sciences and Facility Management's Institute of Natural Resource Sciences.

| **Bacterial species** | **Strain code** | **Geographic origin** | **Reference** |
| --- | --- | --- | --- |
| *Pseudomonas syringae* pv. *actinidiae* (biovar 3) | 27^a^ | PT-Guimarães | - |
|  | AL 2.1d^a^ | PT-Amares | - |
|  | AL 4.2c^a^ | PT-Amares | - |
|  | AL114b^a^ | PT-Amares | Moura et al. (2015) |
|  | AL115^a^ | PT-Amares | Moura et al. (2015) |
|  | AL116b^a^ | PT-Amares | Mariz-Ponte et al. (2022) |
|  | AL13^a^ | PT-Amares | Moura et al. (2015) |
|  | Am 3.1c^a^ | PT-Amarante | - |
|  | AN 1.3a^a^ | PT-Anadia | - |
|  | AN 4.3a^a^ | PT-Anadia | - |
|  | AV 1.3c^a^ | PT-Aveiro | - |
|  | BG 3.1a^a^ | PT-Briteiros | - |
|  | BG 6.1b^a^ | PT-Briteiros | - |
|  | BG 7.2d^a^ | PT-Briteiros | - |
|  | C1.1a^a^ | PT-Celorico de Basto | - |
|  | C2.2a^a^ | PT-Celorico de Basto | - |
|  | CP 1.2c^a^ | PT-Castelo de Paiva | - |
|  | CP 3.1b^a^ | PT-Castelo de Paiva | - |
|  | CP 6.2a^a^ | PT-Castelo de Paiva | - |
|  | CP 7.1b^a^ | PT-Castelo de Paiva | - |
|  | CP 8.1a^a^ | PT-Castelo de Paiva | - |
|  | F 2.3a^a^ | PT-Felgueiras | - |
|  | F1.3B^a^ | PT-Felgueiras | - |
|  | Fv62^a^ | PT-Felgueiras | Moura et al. (2015) |
|  | GM 1.1a^a^ | PT-Guimarães | - |
|  | GM 2.1a^a^ | PT-Guimarães | - |
|  | GM 3.3b^a^ | PT-Guimarães | - |
|  | MV2.3B^a^ | PT-Montemor-o-velho | - |
|  | P84^a^ | PT-Prado | Moura et al. (2015) |
|  | P85^a^ | PT-Prado | Moura et al. (2015) |
|  | P93^a^ | PT-Prado | Moura et al. (2015) |
|  | PB 2.2c^a^ | PT-Pombal | - |
|  | PB 4.2a^a^ | PT-Pombal | - |
|  | Pn16^a^ | PT-Penafiel | Moura et al. (2015) |
|  | SL 2.2b^a^ | PT-Braga | - |
|  | SL 4.2b^a^ | PT-Braga | - |
|  | ST1.1C^a^ | PT-Santo Tirso | - |
|  | VC104b^a^ | PT-Vila do Conde | Moura et al. (2015) |
|  | VN23^a^ | PT-Valença | Mariz-Ponte et al. (2022) |
|  | VN28^a^ | PT-Valença | Mariz-Ponte et al. (2022) |
|  | VV10^a^ | PT-Valença | Mariz-Ponte et al. (2022) |
|  | VV112^a^ | PT-Valença | Moura et al. (2015) |
|  | VV3^a^ | PT-Valença | Mariz-Ponte et al. (2022) |
|  | CFBP7286 ^H,a^ | Italy | Mazzaglia et al. (2012) |
|  | ICMP 18839^c^ | New Zealand | Chapman et al. (2012) |
|  | Jilo20^c^ | China | Ruinelli et al. (2017) |
|  | LNPV36.32^c^ | France | Ruinelli et al. (2017) |
|  | Haxa6^c^ | China | Ruinelli et al. (2017) |
|  | LSV38.17^c^ | China | Ruinelli et al. (2017) |
|  | PSA 490^c^ | Italy | Ruinelli et al. (2017) |
| *Pseudomonas syringae* pv. *actinidiae* (biovar 1) | 2726^c^ | Japan | Ruinelli et al. (2017) |
|  | 2820^c^ | Japan | Ruinelli et al. (2017) |
|  | CFBP 5098^c^ | Japan | Ruinelli et al. (2017) |
|  | Kw41^c^ | Japan | Mazzaglia et al. (2012) |
|  | CFBP4909^c^ | Japan | Marcelletti et al. (2011) |
|  | PA459^c^ | Japan | Mazzaglia et al. (2012) |
| *Pseudomonas syringae* pv. *actinidiae* (biovar 2) | K3^c^ | Korea | Ruinelli et al. (2017) |
|  | KCTC 23663^c^ | Korea | Ruinelli et al. (2017) |
|  | KCTC 23665^c^ | Korea | Ruinelli et al. (2017) |
| *Pseudomonas syringae* pv. *actinidiae* (biovar 4) | ICMP 18804^c^ | New Zealand | - |
|  | ICMP 18882^c^ | New Zealand | Chapman et al. (2012) |
| *P. syringae* pv. *tomato* | DC3000^b^ | United Kingdom | Albuquerque et al. (2012) |
| *P. syringae* pv. *syringae* | Pss10604^b^ | United Kingdom | Albuquerque et al. (2012) |
| *P. syringae* pv. *tabaci* | Pst5393^b^ | Hungary | Albuquerque et al. (2012) |
| *P. cerasi* | B65^a^ | PT-Braga | - |
| *Bacillus safensis* | FV46^a^ | PT-Vila Real | Costa-Santos et al. (2021) |
| *Bacillus subtilis* | UTAD EF2^a^ | PT-Vila Real | - |
| *P. fluorescens* | Pf0-1^a^ | United stated of America | Albuquerque et al. (2012) |
| *P. viridifvala* | CFBP2107^a^ | Switzerland | - |
| *P. putida* | BG2C10C^a^ | Portugal | Correia et al. (2021) |

**Supplementary Table S2. -** Host range of bacteriophage Brt_Psa3. Efficiency of plating (EOP): the same number as in the propagation strain (+++), if is shifted by one level (10x, ++), two levels (+) and no plaques observed (-). (H) bacterial host used for bacteriophage isolation and propagation.

| **Species** | **Strain** | **Origin** | **EOP** |
| --- | --- | --- | --- |
| *Pseudomonas syringae* pv. *actinidiae* (biovar 3) | 27 | PT-Guimarães | +++ |
|  | AL 2.1d | PT-Amares | +++ |
|  | AL 4.2c | PT-Amares | +++ |
|  | AL114b | PT-Amares | ++ |
|  | AL115 | PT-Amares | - |
|  | AL116b | PT-Amares | ++ |
|  | AL13 | PT-Amares | ++ |
|  | Am 3.1c | PT-Amarante | +++ |
|  | AN 1.3a | PT-Anadia | +++ |
|  | AN 4.3a | PT-Anadia | +++ |
|  | AV 1.3c | PT-Aveiro | +++ |
|  | BG 3.1a | PT-Briteiros | +++ |
|  | BG 6.1b | PT-Briteiros | +++ |
|  | BG 7.2d | PT-Briteiros | ++ |
|  | C1.1a | PT-Celorico de Basto | +++ |
|  | C2.2a | PT-Celorico de Basto | ++ |
|  | CP 1.2c | PT-Castelo de Paiva | +++ |
|  | CP 3.1b | PT-Castelo de Paiva | +++ |
|  | CP 6.2a | PT-Castelo de Paiva | ++ |
|  | CP 7.1b | PT-Castelo de Paiva | +++ |
|  | CP 8.1a | PT-Castelo de Paiva | +++ |
|  | F 2.3a | PT-Felgueiras | +++ |
|  | F1.3B | PT-Felgueiras | ++ |
|  | Fv62 | PT-Felgueiras | ++ |
|  | GM 1.1a | PT-Guimarães | ++ |
|  | GM 2.1a | PT-Guimarães | ++ |
|  | GM 3.3b | PT-Guimarães | ++ |
|  | MV2.3B | PT-Montemor-o-velho | ++ |
|  | P84 | PT-Prado | +++ |
|  | P85 | PT-Prado | +++ |
|  | P93 | PT-Prado | +++ |
|  | PB 2.2c | PT-Pombal | ++ |
|  | PB 4.2a | PT-Pombal | ++ |
|  | Pn16 | PT-Penafiel | ++ |
|  | SL 2.2b | PT-Braga | ++ |
|  | SL 4.2b | PT-Braga | +++ |
|  | ST1.1C | PT-Santo Tirso | +++ |
|  | VC104b | PT-Vila do Conde | +++ |
|  | VN23 | PT-Valença | - |
|  | VN28 | PT-Valença | +++ |
|  | VV10 | PT-Valença | ++ |
|  | VV112 | PT-Valença | - |
|  | VV3 | PT-Valença | - |
|  | CFBP7286 ^H^ | Italy | +++ |
|  | ICMP 18839 | New Zealand | ++ |
|  | Jilo20 | China | + |
|  | LNPV36.32 | France | + |
|  | Haxa6 | China | + |
|  | LSV38.17 | China | + |
|  | PSA 490 | Italy | + |
| *Pseudomonas syringae* pv. *actinidiae* (biovar 1) | 2726 | Japan | - |
|  | 2820 | Japan | + |
|  | CFBP 5098 | Japan | - |
|  | Kw41 | Japan | - |
|  | CFBP4909 | Japan | - |
|  | PA459 | Japan | + |
| *Pseudomonas syringae* pv. *actinidiae* (biovar 2) | K3 | Korea | + |
|  | KCTC 23663 | Korea | - |
|  | KCTC 23665 | Korea | + |
| *Pseudomonas syringae* pv. *actinidiae* (biovar 4) | ICMP 18804 | New Zealand | - |
|  | ICMP 18882 | New Zealand | - |
| *Pseudomonas syringae* pv. *tomato* | DC3000 | United Kingdom | ++ |
| *Pseudomonas syringae* pv. *syringae* | Pss10604 | United Kingdom | - |
| *Pseudomonas syringae* pv. *tabaci* | Pst5393 | Hungary | - |
| *Pseudomonas cerasi* | B65 | PT-Braga | +++ |
| *Bacillus safensis* | FV46 | PT-Vila Real | - |
| *Bacillus subtilis* | UTAD EF2 | PT-Vila Real | - |
| *Pseudomonas fluorescens* | Pf0-1 | United stated of America | - |
| *Pseudomonas viridifvala* | CFBP2107 | Switzerland | +++ |
| *Pseudomonas putida* | BG2C10C | Portugal | - |

**Supplementary Table S3** – Predicted ORFs and their functional annotations in the genome of bacteriophage Brt_Psa3.

| **ORF** | **Strand** | **Protein size (bp)** | **E-value (% identify)** | **Putative funclion** | **Best species hit (accession number)** |
| --- | --- | --- | --- | --- | --- |
| 1 | Reverse | 4200 | 0.0 (84.21%) | Internal virion protein D with endolysin domain | *Pseudomonas* phage PPPL-1 (YP_009187987.1) |
| 2 | Reverse | 2679 | 0.0 (88.91) | RNA polymerase | *Pseudomonas* phage PPPL-1 (YP_009187954.1) |
| 3 | Reverse | 2421 | 0.0 (92.68) | Tail protein | *Pseudomonas* phage shl2 (YP_009824198.1) |
| 4 | Reverse | 2190 | 0.0 (85.5) | Internal virion protein C | *Pseudomonas* phage MR2 (A0A6M3T8Y4) |
| 5 | Reverse | 2145 | 0.0 (90.20) | DNA polymerase I | *Pseudomonas* phage shl2 (YP_009824161.1) |
| 6 | Reverse | 1839 | 0.0 (64.16) | Tail fiber protein | *Pseudomonas* phage KNP (YP_009790528.1) |
| 7 | Reverse | 1740 | 0.0 (96.03) | Terminase large subunit | Pseudomonas phage PPPL-1 (YP_009187993.1) |
| 8 | Reverse | 1713 | 0.0 (96.47) | DNA primase/helicase | *Pseudomonas* phage shl2 (YP_009824164.1) |
| 9 | Reverse | 1635 | 0.0 (95.21) | Portal protein, head-tail adaptor protein | *Pseudomonas* phage shl2 (YP_009824202.1) |
| 10 | Reverse | 1068 | 0.0 (89.86) | ATP-dependent DNA ligase | *Pseudomonas* phage Eir4 (UGL61066.1) |
| 11 | Reverse | 1029 | 0.0 (97.36) | Major head protein | *Pseudomonas* phage Henninger (YP_009798001.1) |
| 12 | Reverse | 939 | 0.0 (90.46) | Exonuclease | *Pseudomonas* phage PPPL-1 (YP_009187974.1) |
| 13 | Reverse | 867 | 0.0 (81.31) | Head assembly protein | *Pseudomonas* phage shl2 (YP_009824201.1) |
| 14 | Reverse | 717 | 0.0 (91.03) | Putative single-stranded DNA-binding protein | *Pseudomonas* phage MR2 (QJD54664.1) |
| 15 | Reverse | 705 | 6E-149 (83.5) | Fe2OG dioxygenase domain-containing protein | *Pseudomonas* phage shl2 (A0A160SXQ8) |
| 16 | Reverse | 657 | 1E-125 (81.74) | Putative deoxynucleotide monophosphate kinase | *Pseudomonas* phage MR2 (QJD54661.1) |
| 17 | Reverse | 606 | 9E-102 (74.26) | Putative minor capsid protein | *Pseudomonas* phage MR1 (QJD54628.1) |
| 18 | Reverse | 588 | 4E-122 (88.21) | Tail protein | *Pseudomonas* phage Henninger (YP_009798002.1) |
| 19 | Reverse | 579 | 1E-129 (97.91) | Internal virion protein B | *Pseudomonas* phage shl2 (YP_009824196.1) |
| 20 | Reverse | 576 | 5E-113 (80.63) | Hypothetical protein | *Pseudomonas* phage PPPL-1 (YP_009187951.1) |
| 21 | Reverse | 558 | 5E-122 (93.48) | Putative polymerase | *Pseudomonas* phage MR2 (QJD54667.1) |
| 22 | Reverse | 519 | 9E-49 (56.88) | Hypothetical protein | *Pseudomonas* phage MR2 (QJD54653.1) |
| 23 | Reverse | 507 | 3E-78 (66.28) | Hypothetical protein | *Pseudomonas* phage MR2 (QJD54671.1) |
| 24 | Reverse | 474 | 3E-76 (71.25) | Hypothetical protein | *Pseudomonas* phage MR2 (QJD54649.1) |
| 25 | Reverse | 459 | 2E-84 (80.92) | Putative Rz-like lysis protein | *Pseudomonas* phage MR1 (QJD54639.1) |
| 26 | Reverse | 444 | 1E-100 (95.92) | Putative endonuclease | *Pseudomonas* phage MR1 (QJD54610.1) |
| 27 | Reverse | 441 | 2E-100 (93.84) | Endolysin, N-acetylmuramoyl-L-alanine amidase | *Pseudomonas* phage shl2 (YP_009824166.1) |
| 28 | Reverse | 435 | 3E-78 (79.17) | Internal virion protein A | *Pseudomonas* phage 17A (YP_009948675.1) |
| 29 | Reverse | 423 | 1.1E-85 (86.4) | *N*-Acetyltransferase domain-containing protein | *Pseudomonas* phage Pf1 ERZ-2017 (A0A2H4YGB0) |
| 30 | Reverse | 360 | 3E-67 (85.71) | Tail fiber protein | *Pseudomonas* phage KNP (YP_009790529.1) |
| 31 | Reverse | 360 | 1E-39 (61.60) | Hypothetical protein | *Pseudomonas* phage 17A (YP_009948700.1) |
| 32 | Reverse | 354 | 1E-55 (84.31) | Gp5.5-like host HNS inhibition | *Pseudomonas* phage Pf1 ERZ-2017 (YP_009793760.1) |
| 33 | Reverse | 348 | 4E-61 (81.03) | Hypothetical protein | *Pseudomonas* phage shl2 (YP_009824169.1) |
| 34 | Reverse | 306 | 4E-33 (85.29) | Host range and adsorption protein | *Pseudomonas* phage shl2 (YP_009824203.1) |
| 35 | Reverse | 285 | 8E-45 (85.26) | Hypothetical protein | *Pseudomonas* phage AH05 (QZA71342.1) |
| 36 | Reverse | 270 | 3E-10 (66) | Hypothetical protein | *Pseudomonas* phage MR2 (A0A6M3T907) |
| 37 | Reverse | 258 | 2E-50 (96.47) | Terminase small subunit | *Pseudomonas* phage PPPL-1 (YP_009187991.1) |
| 38 | Reverse | 249 | 2E-43 (85.37) | Hypothetical protein | *Pseudomonas* phage PPPL-1 (YP_009187946.1) |
| 39 | Reverse | 243 | 1E-43 (87.50) | Hypothetical protein | *Pseudomonas* phage MR2 (QJD54677.1) |
| 40 | Reverse | 243 | 1E-45 (89.87) | Hypothetical protein | *Pseudomonas* phage AH05 (QZA71318.1) |
| 41 | Reverse | 231 | 4E-33 (81.58) | Hypothetical protein | *Pseudomonas* phage MR2 (QJD54670.1) |
| 42 | Reverse | 225 | 1E-31 (94.37) | Holin | *Pseudomonas* phage PPPL-1 (YP_009187990.1) |
| 43 | Reverse | 213 | 3E-31 (75.71) | Hypothetical protein | *Pseudomonas* phage MR1 (QJD54641.1) |
| 44 | Reverse | 210 | 3E-31 (75.71) | Hypothetical protein | *Pseudomonas* phage MR1 (QJD54641.1) |
| 45 | Reverse | 207 | 6.2E-43(100) | HNS binding protein | *Pseudomonas* phage shl2 (A0A160SW93) |
| 46 | Reverse | 198 | 4E-35 (86.76) | Hypothetical protein | *Pseudomonas* phage shl2 (YP_009824180.1) |
| 47 | Reverse | 174 | 3E-25 (80.70) | Hypothetical protein | *Pseudomonas* phage MR2 (QJD54660.1) |
| 48 | Reverse | 171 | 6E-30 (94.64) | RNA polymerase inhibitor | *Pseudomonas* phage PPPL-1 (YP_009187962.1) |
| 49 | Reverse | 141 | 0.009 (74.07) | Hypothetical protein | *Pseudomonas* phage MR1 (QJD54642.1) |
| 50 | Reverse | 138 | 4E-14 (83.33) | Putative DNA-directed RNA polymerase | *Pseudomonas* phage MR2 (QJD54655.1) |
| 51 | Reverse | 129 | 9E-10 (82.76) | Putative virion structural protein | *Pseudomonas* phage MR1 (QJD54604.1) |

**Supplementary Figure S1** – Phylogenetic analysis of bacteriophage Brt_Psa3 based on a) the amino acid sequences of terminase large subunit and b) the major capsid protein.


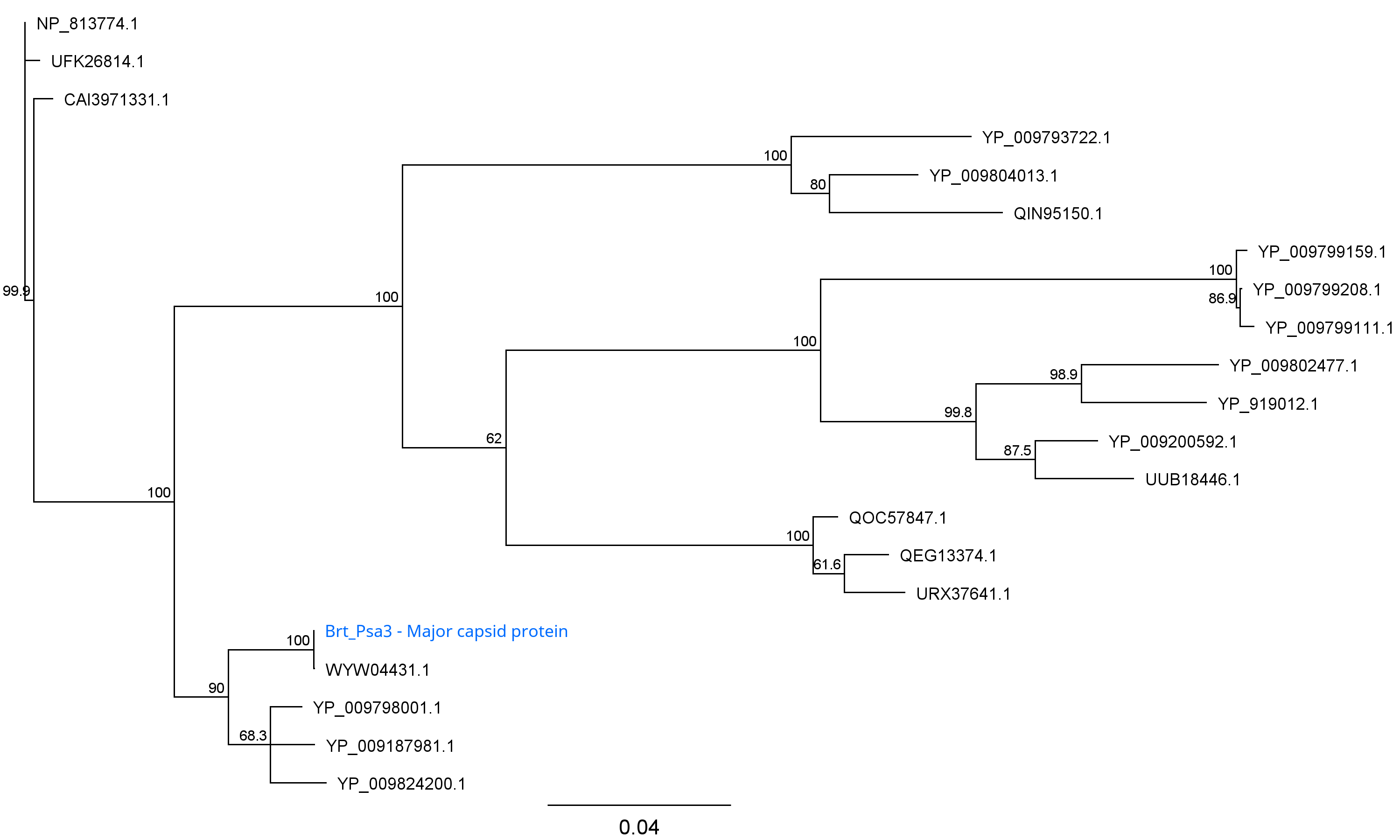

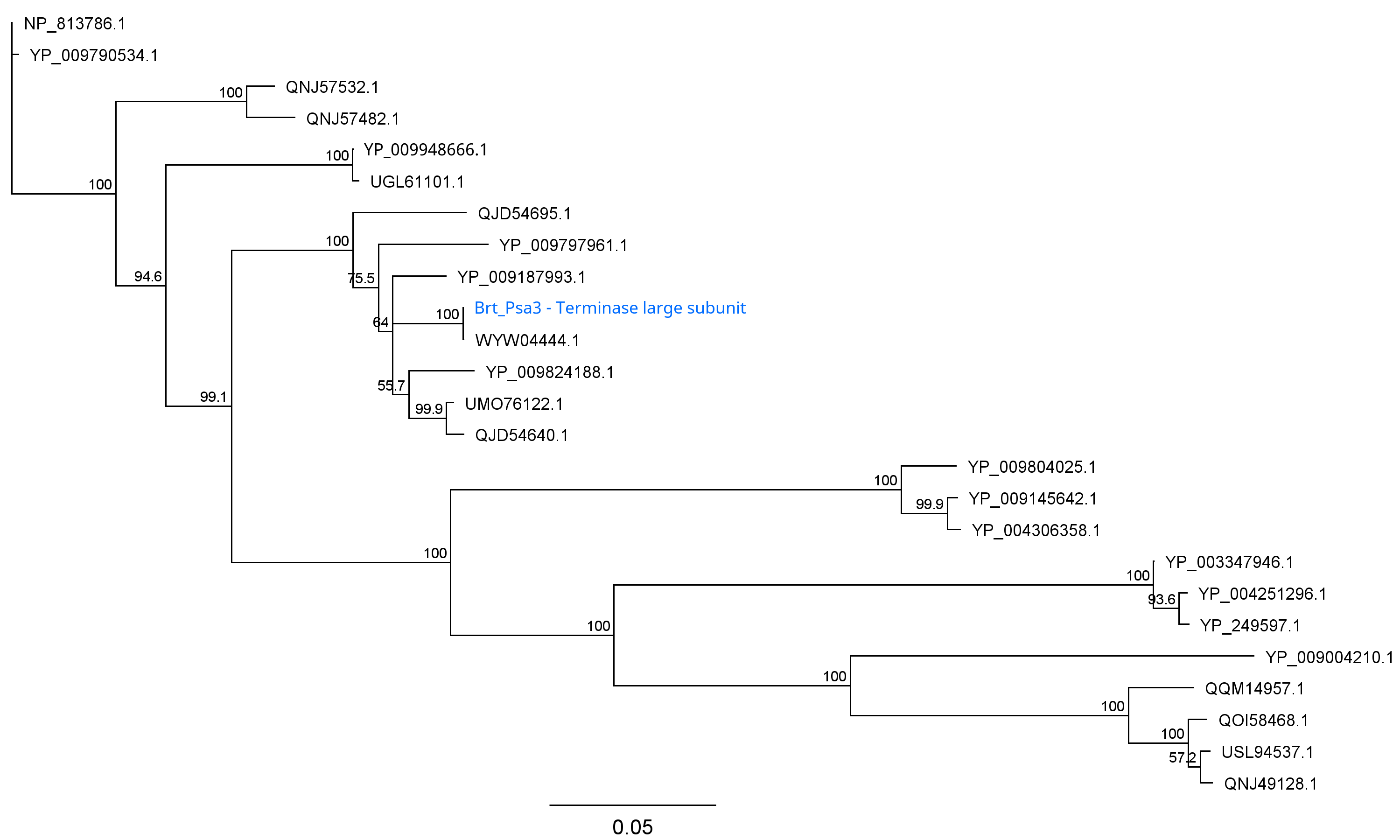


**a)**

**b)**

*Studiervirinae, Ghunavirus*

*Studiervirinae, Ghunavirus*

*Studiervirinae, Ghunavirus*

*Studiervirinae, Pifdecavirus*

*Studiervirinae, Pifdecavirus*

*Studiervirinae, Chatterjeevirus*

*Studiervirinae, Caroctavirus*

*Studiervirinae, Kayfunavirus*

*Studiervirinae, Helsettvirus*

*Studiervirinae, Ningirsuvirus*

*Studiervirinae, Berlinvirus*

**References**

Albuquerque P, Caridade CMR, Rodrigues AS, Marcal ARS, Cruz J, Cruz L, Santos CL, Mendes MV, Tavares F (2012) Evolutionary and experimental assessment of novel markers for detection of *Xanthomonas euvesicatoria* in plant samples. PLoS ONE 7:e37836. doi: 10.1371/journal.pone.0037836

Chapman JR, Taylor RK, Weir BS, Romberg MK, Vanneste JL, Luck J, Alexander BJR (2012) Phylogenetic relationships among global populations of *Pseudomonas syringae* pv. *actinidiae*. Phytopathol 102:1034-1044. doi: 10.1094/PHYTO-03-12-0064-R

Correia CV, Mariz da Ponte NA, Cellini A, Donati I, Santos C, Spinelli F (2022) Selection of biological control agents against the pathogen *Pseudomonas syringae* pv. *actinidiae* from phyllosphere of kiwifruit leaves. Acta Hortic 1332:117-124. doi: 10.17660/ActaHortic.2022.1332.16

Mariz-Ponte N, Gimranov E, Rego R, Moura L, Santos C, Tavares F (2022) Distinct phenotypic behaviours within a clonal population of *Pseudomonas* *syringae* pv. *actinidiae*. PLoS ONE 17:e0269343. doi: 10.1371/journal.pone.0269343

Mazzaglia A, Studholme DJ, Taratufolo MC, Cai R, Almeida NF, Goodman T, Guttman DS, Vinatzer BA, Balestra GM (2012) *Pseudomonas syringae* pv. *actinidiae* (PSA) isolates from recent bacterial canker of kiwifruit outbreaks belong to the same genetic lineage. PLoS ONE 7:e36518. doi: 10.1371/journal.pone.0036518

Moura L, Garcia E, Aguín O, Ares A, Abelleira A, Mansilla P (2015) Identificação e caracterização de *Pseudomonas syringae* pv. *actinidiae* (Psa) na Região do Entre Douro e Minho (Portugal). Revista de Ciências Agrárias 38:196-205. doi: 10.19084/rca.16915

Ruinelli M, Schneeberger PH, Ferrante P, Bühlmann A, Scortichini M, Vanneste JL, Duffy B, Pothier JF (2017) Comparative genomics‐informed design of two LAMP assays for detection of the kiwifruit pathogen *Pseudomonas* *syringae* pv. *actinidiae* and discrimination of isolates belonging to the pandemic biovar 3. Plant Pathol 66:140-149. doi: 10.1111/ppa.12551
